# Association of Social Anxiety Disorder and Physical Activity With Psychological Distress During COVID-19 Imposed Lockdown: A Medical Student Survey From a Developing Country

**DOI:** 10.1192/bjo.2022.169

**Published:** 2022-06-20

**Authors:** Muhammad Arish, Muhammad Arham, Fatima Rehan, Sajeel Saeed, Kashif Tousif, Muhammad Farhan

**Affiliations:** Rawalpindi Medical University, Rawalpindi, Pakistan

## Abstract

**Aims:**

COVID-19 imposed lockdowns have impacted the mental health of medical students worldwide. However, the relationship of social anxiety disorder with psychological distress during the pandemic has not been studied. The objective of this study was to assess the association of social anxiety disorder and physical activity with psychological distress in medical students during COVID-19 imposed lockdown.

**Methods:**

256 medical students (M/F: 144/112) took part in this online cross-sectional survey conducted in September 2020 during a government imposed lockdown. Kessler-10 (K10) questionnaire, social interaction anxiety scale (SIAS) and international physical activity questionnaire (IPAQ) - short form were used to assess psychological distress, social anxiety and physical activity, respectively. Data were analysed using SPSS v25, with application of multivariate logistic regression to assess association of various factors with psychological distress.

**Results:**

Out of 256 medical students, 73 (28.5%) had severe psychological distress and 105 (41.0%) had mild to moderate distress. A logistic regression model to assess the effect of social anxiety disorder, level of physical activity and gender with the likelihood of having severe psychological distress was statistically significant (p < 0.001) with overall accuracy of 73.8%. The risk of developing severe psychological distress was higher among females [OR 2.13 (95% CI 1.17–3.87), p = 0.013] and those with social anxiety disorder [OR 4.56 (95% CI 2.27–9.16), p < 0.001]. Low physical activity was not a significant risk factor for psychological distress [OR 0.88 (95% CI 0.35–2.23), p = 0.794].

**Conclusion:**

This study shows that COVID-19 imposed lockdown has adversely affected the mental health of medical students. Female students and those with social anxiety disorder are at more risk of developing severe psychological distress.

